# Nerve Growth Factor Modulates Regulatory Cell Volume Behavior via Stimulating TRPV1, TRPM8 Channels and Inducing Ca^2+^ Signaling in Human Conjunctival Epithelial Cells

**DOI:** 10.3390/cells14100719

**Published:** 2025-05-15

**Authors:** Friedrich Wolf, Tina Dietrich-Ntoukas, Peter S. Reinach, Uwe Pleyer, Stefan Mergler

**Affiliations:** 1 Department of Ophthalmology, Charité—Universitätsmedizin Berlin, Campus Virchow-Klinikum, Augustenburger Platz 1, 13353 Berlin, Germany; friedrich.wolf@charite.de (F.W.); tina.dietrich-ntoukas@charite.de (T.D.-N.); uwe.pleyer@charite.de (U.P.); 2 School of Ophthalmology and Optometry, Wenzhou Medical University, Wenzhou 325015, China; preinach25@gmail.com

**Keywords:** nerve growth factor, TRPV 1, TRPM 8, conjunctival epithelial cells, Ca^2+^ regulation

## Abstract

NGF plays important roles in ocular surface homeostasis and different pathological conditions. One effect includes promoting conjunctival epithelial cell differentiation and mucin secretion. This study characterizes the individual roles of TRPV1 and TRPM8 channel activity in mediating the effects of NGF on intracellular Ca^2+^ regulation and its alteration of regulatory cell volume responses to anisosmotic challenges in human conjunctival epithelial cells (IOBA-NHC). With fura-2/AM-loaded cells, the effects of 40 µM capsaicin and 20 µM AMG 9810 on Ca^2+^ regulation confirm functional TRPV1 expression. TRPM8 expression is evident since 500 µM menthol and 20 µM AMTB have opposing effects on [Ca^2+^]_i_. AMG 9810 and AMTB (both 20 µM) suppress the responses to NGF (100 ng/mL). With calcein/AM-loaded cells, the effects of these mediators are evaluated on apparent cell volume responses induced by an anisosmotic challenge. NGF decreases the apparent cell volume that AMG 9810 suppresses, whereas AMTB (both 20 µM) augments this response. Therefore, NGF interacts with TRPV1 and TRPM8 to induce opposing effects on cell volume regulatory behavior. These opposing effects suggest that the signaling pathways and effectors that mediate responses to TRPV1 and TRPM8 activation are not the same.

## 1. Introduction

The conjunctiva and the eyelids, along with the lacrimal glands, perform nutritive and defensive tasks on the surface of the eye. This complex is integrated into a dense neuronal and immunological network called the “Conjunctiva-Associated Lymphoid Tissue” (CALT) [1,2]. This syncytium perceives different stimuli and on the one hand also minimizes collateral damage. In addition, resident microorganisms colonize the conjunctiva (microbiome), and numerous biophysical factors can disturb the ocular surface homeostasis.

A critical component of ocular surface homeostasis is the tear film. If compromised or poorly formed, inflammation, and dry eye disease can develop (keratoconjunctivitis sicca) [3,4]. The tear film is composed of three major layers: a lower mucin layer, secreted by goblet cells located within the conjunctiva; an aqueous layer, produced by the accessory glands; and a lipid layer, which is produced by meibomian glands to protect against evaporation [5]. In the case of dry eye disease, the tear film may lose volume due to pathological conditions or evaporate too quickly, which dries out the ocular surface [6,7,8]. Additionally, the hyperosmolarity of the tear film has been demonstrated to cause damage to the ocular surface cells [9].

Corneal epithelial cells express nerve growth factor (NGF) and a cognate receptor. Their expression rises when this outermost syncytial cell layer is damaged. This response indicates that NGF is essential for mediating upregulation of tissue cell migration and proliferation following injury. This cytokine also increases inflammatory processes through immune cell infiltration and activation [10,11,12,13,14,15,16] in the conjunctival goblet cells [17]. Its main receptor is the tropomyosin receptor, which has the highest affinity for NGF [18,19]. NGF affects these downstream events through activating calcium signaling pathways, and transcription via TrkA, which is an auto phosphorylating receptor inducing activation of numerous downstream signaling pathways. The lower affinity neurotrophin receptor P75 (NTR) is also an NGF receptor. This receptor activates TrkA and a signaling cascade that leads to the activation of ERK1/2 and AKT [20], followed by stimulation of the NF-κB- and the Janus kinase-linked pathways [21,22]. However, there are no studies describing how at the cell membrane level receptor activation induces these downstream signaling pathways to stimulate transcription factor activity.

Ca^2+^ signaling triggers the control of innumerable physiological responses. Its homeostasis is regulated by different types of cell membrane delimited ion channels that include voltage dependent, receptor-mediated routes, the transient receptor potential channels (TRP’s) [23], as well as the calcium ATPase ion pumps and the sodium-calcium exchanger (NCX) [20,24]. The SERCA (sarcoplasmic and endoplasmic reticulum calcium ATPase) pump extrudes Ca^2+^ from the endoplasmic reticulum in response to activation of a plasma membrane store-operated Ca^2+^ channel (SOC) [25]. The conjunctiva expresses non-excitable TRP channels [26]. TRPs are divided into six subgroups consisting of four subunits with six transmembrane domains. They are non-selective cation channels, that gate monovalent and divalent cations, and they are able to respond to various stimuli such as heat, cold, taste, pressure, or chemical ligands [27,28,29]. In addition, some of them respond to pain and itch [28,30,31,32] and changes in osmolarity [33]. The capsaicin receptor TRPV1 acts as a sensor of hyperosmolarity, nociception, thermosensation, and chemesthesis [34,35,36]. On the other hand, TRPM8 is a receptor for cold (<25 °C), and menthol and icilin are agonists [37,38,39,40,41]. TRPM8 is also a neuronal osmosensor that regulates eye blinking in mice [42]. In dry eye disease, both TRPV1and TRPM8 modulate this condition [26,34,43,44,45,46]. TRPV1 activation by a hyperosmolar stress mediates cell volume shrinkage in the conjunctival epithelial cell line IOBA-NHC (Instituto de OftalmoBiologia Aplicada-Normal Human Conjunctiva) [47]. TRPM8 is involved in the tear fluid production and is activated in dry eye disease [46,48].

This study was undertaken to probe for crosstalk between TRPV1 and TRPM8 in mediating responses to NGF through modulating calcium regulation, as well as the cell volume behavior induced in IOBA-NHC cells. The results from this study may help improve our understanding of the pathophysiological processes underlying eye diseases and may identify possible therapeutic strategies in ocular surface disease.

## 2. Materials and Methods

Cell culture medium and other cell culture supplements were purchased from Biochrom AG (Berlin, Germany) or GIBCO Invitrogen (Karlsruhe, Germany). Human recombinant beta-NGF was obtained from Biogems company (Westlake Village, CA, USA). AMG 9810, AMTB, CAP and CPZ were obtained from Cayman Chemical Company (Ann Arbor, MI, USA). (+) Menthol was bought from Sigma-Aldrich (Deisenhofen, Germany). Fura2/AM and calcein/AM were ordered from Cayman Chemical Company (Ann Arbor, MI, USA).

### 2.1. Solutions

The measuring solution contained 150 mM NaCl, 6 mM CsCl, 1.5 mM CaCl_2_, 1 mM MgCl_2_, 10 mM glucose, and 10 mM HEPES at 317 mosmol/L and pH 7.4 (Ringer-like solution; RLS). The Ca^2+^-free solution is identical to the basic RLS, but without CaCl_2_ and with an additional Ca^2+^ buffer (1 mM EGTA) as previously described [47]. The isotonic solution for the hypotonic experiments includes 105 mM NaCl, 6 mM CsCl, 1 mM MgCl_2_, 1.5 mM CaCl_2_, 10 mM glucose, 10 mM HEPES-acid, and 80 mM D-mannitol (pH: 7.4; osmolarity: 313 mosmol/L); the hypotonic solution includes 105 mM NaCl, 6 mM CsCl, 1 mM MgCl_2_, 1.5 mM CaCl_2_, 10 mM glucose, and 10 mM HEPES-acid (pH: 7,4; osmolarity: 233 mosmol/L). In the hypertonic experiment, an isotonic solution was used consisting of 105 mM NaCl, 6 mM CsCl, 1 mM MgCl_2_, 1.5 mM CaCl_2_, 10 mM glucose, and 10 mM HEPES-acid (pH: 7,4; osmolarity: 232,88 mosmol/L) and a hypertonic solution of 150 mM NaCl, 6 mM CsCl, 1 mM MgCl_2_, 1.5 mM CaCl_2_, 10 mM glucose, 10 mM HEPES-acid, and 130 mM D-mannitol (pH: 7,4; osmolarity: 447 mosmol/L) [49].

### 2.2. IOBA-NHC Cell Cultivation

The IOBA-NHC (Instituto de OftalmoBiologia Aplicada-Normal Human Conjunctiva) cell line is a spontaneously immortalized cell line developed by Y. Diebold et al. in 2003 [50]. This established model was provided by Friedrich Paulsen and Fabian Garreis (Erlangen, Germany) [50]. Cells were cultivated as previously described [26,47]. In brief, the cells were cultivated at 37 °C with 5% CO_2_. The medium contained 1:1 DMEM/HAM’s F-12 medium, including 10% FBS, penicillin/streptomycin, insulin, and hydrocortisone [26,50].

### 2.3. Fura-2 Fluorescence Calcium Imaging

The single-cell fura-2 fluorescence calcium imaging method was used as recently described [51]. Fura-2 as a ratiometric, calcium-sensitive dye changes its fluorescence excitation spectrum when it binds to calcium. The dye is excited at 340 nm (when calcium is bound) and 380 nm (when calcium is unbound). The emission is typically measured at a fixed wavelength (≈510 nm) [52]. The ratio of fluorescence intensities at 340/380 nm excitation (f340/f380) is an index of the intracellular calcium concentration. This ratio is independent of dye concentration, photobleaching, and cell thickness. Cells were set on coverslips (ø: 15 mm) in a 12-well plate and were then loaded with 1 µM fura-2/AM [52] together with RLS in a darkened room at 37 °C for 20–40 min. Cells were washed in RLS to stop the fluorescent staining. The photometry setup was equipped with a fluorescence microscope (Olympus BW50WI, Europa Holding GmbH, Hamburg, Germany) and a software-controlled high-powered fluorescence LED light source by (Omikron V.1.0, Rodgau-Dudenhoven, Germany), as well as a black and white digital camera (Olympus XM10, Olympus, Hamburg, Germany). This was connected with the U-RTC-Real-Time Controller (Olympus Europa Holding GmbH, Hamburg, Germany) with a Windows PC and controlled over cellSens Dimension V. 1.16 software (Olympus Europa Holding GmbH, Hamburg, Germany). The f340/f380 ratio was calculated for each cell at a region of interest (ROI using the cellSens software. The fluorescence ratio (f340/f380) proportional to intracellular calcium concentration was calculated using cellSens software [52,53]. The fluorescence ratios were normalized (control set to 0.1) and drift-corrected using TIDA software V. 5.25 for Windows (HEKA Electronic, Lamprecht, Germany).

### 2.4. Calcein Fluorescence Cell Volume Imaging

For the single-cell calcein cell volume imaging, measurements were carried out as previously published using the same setup and software [54]. The hypotonic and the hypertonic challenges were performed with the above-mentioned isotonic solution for each experiment (chapter 4.1). An Olympus BX50WI fluorescence microscope in conjunction with a XM10 digital camera (both Olympus, Europa Holding GmbH, Hamburg, Germany) monitored the fluorescence emission of calcein-loaded IOBA-NHC cells. The cells were loaded with 1 µmol/L calcein/AM (Cayman, Ann Arbor, MI, USA) in the growth medium at 37 °C in an incubator with 5% CO_2_ for 30–40 min. Calcein fluorescence excitation wavelength at 494 nm and emission at 517 nm were measured using an ultra-bandpass filter set (500/534 nm bandpass). A LED-HUB with a 505 nm high power LED and a peak wavelength at 495 nm was used as the light source (Omikron, Rodgau-Dudenhofen, Germany). After rinsing with an isotonic RLS (313 mosmol/L), the coverslips were again superfused in the same RLS in the bath chamber (control) for 4 min. The aforementioned hyper- and hypotonic solutions were then applied in the presence or absence of NGF, AMG, and AMTB.

### 2.5. Statistical Data Analyses

A normality test was carried out to check whether the data had a Gaussian distribution or were normally distributed (Kolmogorov–Smirnov test). If the normality test failed, the non-parametric Wilcoxon matched-pairs test was used otherwise the parametric Student’s *t*-test was used. For unpaired data failing the normality test, the non-parametric Mann–Whitney U test was used. Only *p* values < 0.05 (specified by asterisks for paired data (*) and hashtags (#) for unpaired data) were considered significant. SigmaPlot version 12.5 for Windows (Systat Software, Inc., Point Richmond, CA, USA) was used to perform statistical tests and diagrams. Column charts were created with the GraphPad Prism software version 5.00 for Windows (La Jolla, CA, USA). The number of cells is bracketed in each figure near the traces or columns. Each experiment employed three different coverslips, and they contained between 20 to 30 cells. All numbers are given as means ± standard error of the mean (SEM) (error bars in both directions).

## 3. Results

### 3.1. TRPV1 and TRPM8 Regulate Intracellular Ca^2+^ in IOBA-NHC Cells

**Figure 1:**
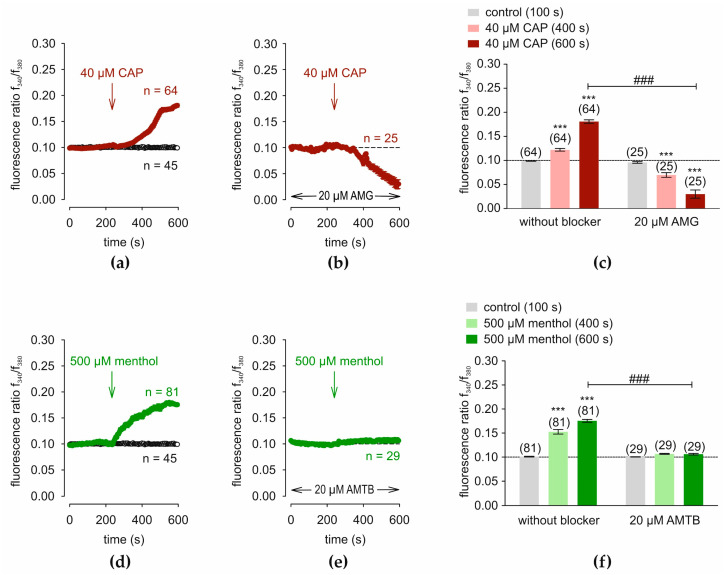
Capsaicin (CAP) and menthol induce Ca^2+^ influx that AMG 9810 (AMG) (TRPV1) and AMTB (TRPM8) suppress, respectively, in fura-2-loaded IOBA-NHC cells. Data are means ± SEM. The number (*n*) above the columns is the number of cells in a corresponding series of experiments. The baseline value is shown by a dashed line (0.1). The arrows show the time (240 s) at which the agonists were added. (**a**) Addition of 40 µM CAP increased [Ca^2+^]_i_ (red filled circles; (*n =* 64). For the control (without CAP), no changes in the [Ca^2+^]_i_ level occurred (*n =* 45) (open circles). (**b**) AMG (20 µM; *n =* 25) inhibited the effect of CAP below the baseline level. (**c**) Summary represents the mean values ± SEM of the fluorescence intensity at 100 s (control) (gray column), 400 s (CAP) (bright red column), and 600 s (CAP) (dark red column). The asterisks (*) indicate statistically significant differences with and without CAP (*n =* 25 to *n =* 64; *** *p* < 0.001; paired tested). The hashtags (#) denote unpaired data with and without AMG (### *p* < 0.001). (**d**–**f**): Same experiments as shown in (**a**–**c**) are performed. 500 µM menthol is used (green filled circles or columns; *n =* 81) and 20 µM AMTB is added (*n =* 29).

TRPV1 and TRPM8 functional expressions were verified in the human conjunctival epithelial cell line IOBA-NHC. Before applying the drugs, control measurements were conducted using fura-2/AM dye. Figure 1a (open circles) shows control measurements (baseline) obtained with Ringer-like solution (RLS) applied for 10 min (*n* = 45). The TRPV1 agonist capsaicin (CAP) (40 µM) [55] and the antagonist AMG 9810 (AMG) (20 µM) [56] were applied. CAP increased the f340 nm/f380 nm fluorescence ratio from 0.0986 ± 0.0004 at 100 s (control) to 0.1221 ± 0.0029 at 400 s and to 0.1808 ± 0.0036 at 600 s (*n* = 64; *** *p* < 0.001; paired tested) (Figure 1a). Following incubation with 20 µM AMG for 20–40 min, the Ca^2+^ increase was suppressed below the baseline level (short dash line) (Figure 1b). The fluorescence ratio, f340/f380, decreased to 0.0698 ± 0.0048 at 400 s and to 0.0300 ± 0.0085 at 600 s, respectively (*n* = 25; *** *p* < 0.001; paired tested) (Figure 1c). Subsequently, the TRPM8 agonist menthol (500 µM) and the TRPM8 antagonist AMTB (20 µM) probed for TRPM8 expression [40,57,58]. Figure 1d shows that menthol increased this ratio from 0.1010 ± 0.0007 at 100 s (control) to 0.1527 ± 0.0048 at 400 s and to 0.1755 ± 0.0031 at 600 s (*n* = 81; *** *p* < 0.001; paired tested). AMTB suppressed these rises to 0.1067 ± 0.0010 at 400 s and to 0.1062 ± 0.0017 at 600 s, respectively (*n* = 29; *** *p* < 0.001; paired tested) (Figure 1e,f). The latter value was also significantly lower than the positive control value with menthol (*n* = 29–81; ### *p* < 0.001) (Figure 1f). Overall, functional TRPV1 and TRPM8 expression was confirmed in IOBA-NHC cells.

### 3.2. NGF-Induces Ca^2+^ Increase via TRPV1 and TRPM8

**Figure 2:**
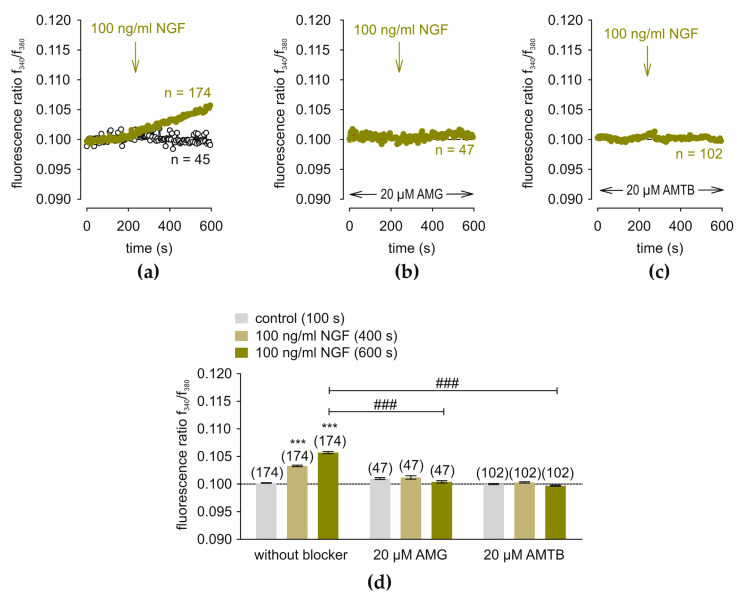
AMG and AMTB suppress NGF-induced Ca^2+^ rises. NGF is present from 240 s (arrow). (**a**) 100 ng/mL increases Ca^2+^ entry (*n* = 174; dark yellow filled circles) in RLS. Control values are shown with open circles (*n* = 45). (**b**) 20 µM AMG fails to alter the NGF-induced Ca^2+^ influx (negative control) (*n* = 47) (**c**) AMTB completely blocks the NGF-induced Ca^2+^ increase (*n* = 102). (**d**) Either AMG or AMTB (both 20 µM) completely block NGF- induced Ca^2+^ response behavior. Columns represent mean values ± SEM of the fluorescence ratio at 100 s (control) (gray column), 400 s (NGF) (yellow column) and 600 s (NGF) (dark yellow column). The asterisks (*) indicate statistically significant differences with and without NGF (*n* = 174; *** *p* < 0.001; paired tested). The hashtags (#) denote unpaired data with and without AMG or AMTB (*n* = 47–102; ### *p* < 0.001; unpaired tested).

Figure 2a shows that both 100 and 250 ng/mL NGF irreversibly increased the f340/f380 fluorescence ratio from 0.1002 ± 0.00004 at 100 s (control) to 0.1033 ± 0.0001 at 400 s to 0.1057 ± 0.0002 at 600 s (*n* = 174; *** *p* < 0.001; paired tested). Both AMG and AMTB abolished this response (AMG: f340/f380 = 0.1012 ± 0.0003 at 400 s and 0.1004 ± 0.0002 at 600 s; *n* = 47; AMTB: f340/f380 = 0.1003 ± 0.0001 at 400 s and 0.0997 ± 0.0001 at 600 s; *n* = 102; both ### *p* < 0.001; unpaired tested) (Figure 2b–d). Two-hundred-and-fifty ng/mL NGF slightly increased the f340/f380 fluorescence ratio (*n* = 20; ## *p* < 0.01; unpaired tested) (Figure S1). Therefore, NGF elicits Ca^2+^ transients through joint activation of TRPV1 and TRPM8.

### 3.3. NGF-Induces Ca^2+^ Influx After Passive Store Depletion

**Figure 3:**
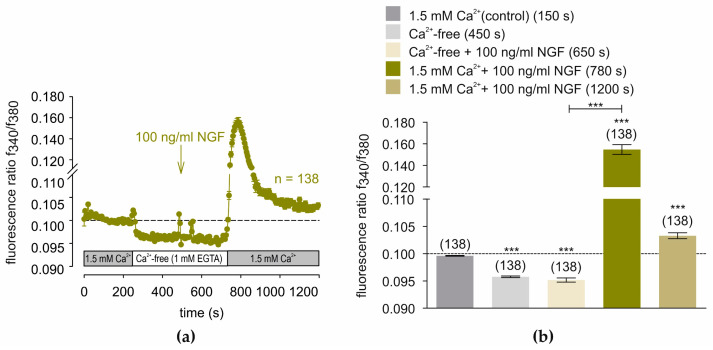
Augmentation of NGF- induced Ca^2+^ transients following store depletion in RLS. From 240 s, the solution was changed from a 1.5 mM Ca^2+^-containing solution (gray bar below the trace) to a Ca^2+^-free solution with 1 mM EGTA (white bar below the trace). NGF is added from 480 s (arrow). From 720 s, the Ca^2+^-free solution is replaced with the control solution containing 1.5 mM Ca^2+^ (gray bar below the trace). (**a**) In the absence of extracellular Ca^2+^, Ca^2+^ decreases below the baseline level, which 100 ng/mL NGF addition had no effect. However, in the presence of NGF following replacement with external 1.5 mM Ca^2+^ (RLS), a large Ca^2+^ transient is generated (*n* = 138, dark yellow filled circles). (**b**) Summary of the experiments with NGF in IOBA-NHC cells with and without extracellular Ca^2+^. The asterisks (***) designate a significant decrease in Ca^2+^-free RLS with and without NGF (*t* = 450 s, 650 s; *n* = 138; *** *p* < 0.001; paired tested) (*t* = 150 s), as well as an increase in Ca^2+^ in the presence of extracellular Ca^2+^ after NGF application (*t* = 780 s, 1200 s; *n* = 138; *** *p* < 0.001; paired tested) (*t* = 150 s).

Figure 3a shows the consecutive effects on NGF-induced [Ca^2+^]_i_ responses at 240 s of Ca^2+^ removal from RLS, and its replacement with a Ca^2+^-free solution (1 mM EGTA). Initially, intracellular Ca^2+^ concentration fell from 0.09961 ± 0.00007 at 150 s (control) to 0.09577 ± 0.00014 at 450 s in the Ca^2+^-free solution (1 mM EGTA) (*n* = 138; *** *p* < 0.001; paired tested). At 480 s, 100 ng/mL NGF was added, but failed to change the f340/f380 fluorescence ratio (f340/f380 = 0.09516 ± 0.00038; *n* = 138; *p* > 0.05; paired tested). At 720 s, replacement was performed with RLS containing 1.5 mM extracellular calcium following passive store depletion. NGF instead induced a large f340/f380 fluorescence ratio increase at 150 s from 0.10330 ± 0.00055 to 0.15470 ± 0.00451 that reclined to 0.10330 ± 0.00055 at 20 min (both *n* = 138; *t* = 1200 s; *** *p* < 0.001; paired tested) (Figure 3b). In summary, NGF induces larger Ca^2+^ responses provided the intracellular Ca^2+^ stores were depleted, and Ca^2+^ was present in the external solution.

### 3.4. Hyper- and Hypotonicity-Induce Opposite Changes in Regulatory Cell Volume

**Figure 4:**
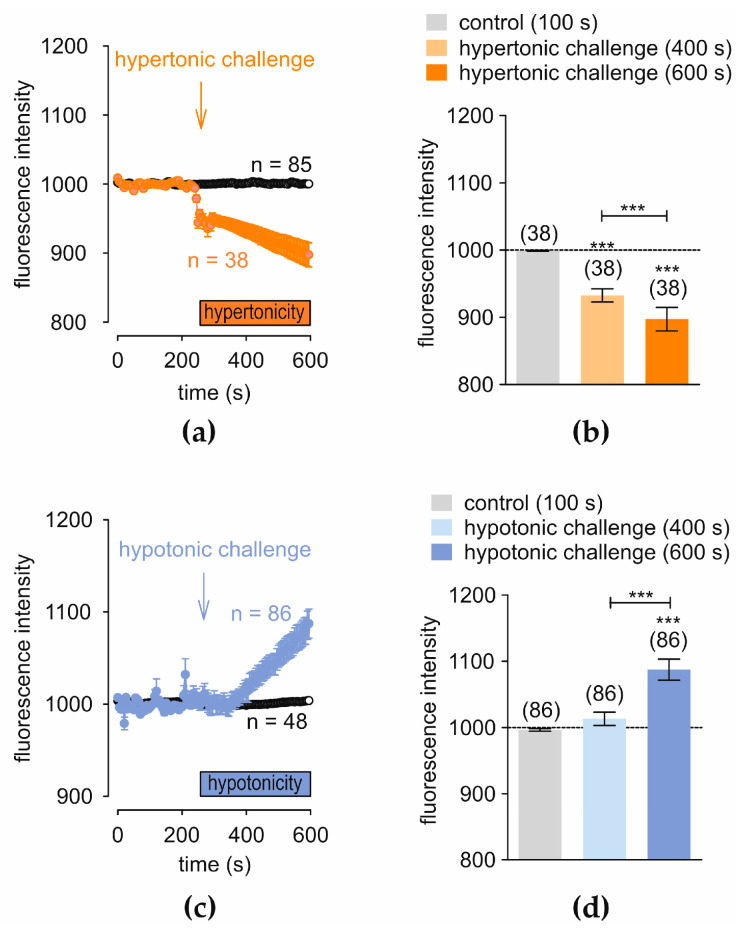
Hypertonicity induces cell shrinkage, whereas hypotonicity induces cell-swelling. The reference line (dashed line) for baseline f494 fluorescence intensity (1000). The arrows mark the time (240 s) at which the osmolarity is changed. (**a**) A hypertonic challenge (450 mosmol/L) reduces fluorescence intensity, (*n* = 38, red filled circles). Isotonic RLS (313 mosmol/L) does not change the fluorescence intensity (*n* = 85; open circles). (**b**) Summary is shown with the hypertonic challenge. Columns represent mean values ± SEM of the fluorescence intensity at 100 (control), 400, and 600 s (hypertonic challenge). The asterisks (*) indicate statistically significant differences with and without hypertonic challenge (*n =* 38; *** *p* < 0.001; paired tested). (**c**) Same experimental sequence as in (**a**), but with hypotonic solution (233 mosmol/L) (blue filled circles) (*n* = 86; *** *p* < 0.001; paired tested). (**d**) Same analyses as shown in (**b**), but with hypotonic solution (blue columns).

Under control conditions, an isotonic RLS (≈313 mosmol/L) was firstly applied for 10 min (*n* = 85) to calcein-loaded IOBA-NHC cells (Figure 4a, open circles). After 4 min, a reduction in the apparent cell volume following exposure to the hypertonic challenge (≈450 mosmol/L) is shown (Figure 4a), which induced a shrinkage. The f494 fluorescence intensity decreased from 999.0665 ± 0.5518 at 100 s (control) to 932.535 ± 9.6773 at 400 s and to 897.2916 ± 17.4347 at 600 s (*n* = 38; *** *p* < 0.001; paired tested) (Figure 4b). A control measurement with RLS was carried out before replacement with hypotonic solution (233 mosmol/L) (*n* = 48) (Figure 4c, open circles). The f494 fluorescence intensity increased with a slight delay from 996.4032 ± 2.0867 at 100 s to 1013.201 ± 10.0303 at 400 s and to 1087.3590 ± 15.7454 at 600 s (*n* = 86; *** *p* < 0.001; paired tested) (Figure 4d). In conclusion, IOBA-NHC cells shrank in the presence of extracellular hyperosmolarity, whereas cell swelling occurred in a hypotonic solution.

### 3.5. TRPV1 and TRPM8 Regulate Cell Volume in IOBA-NHC Cells

**Figure 5:**
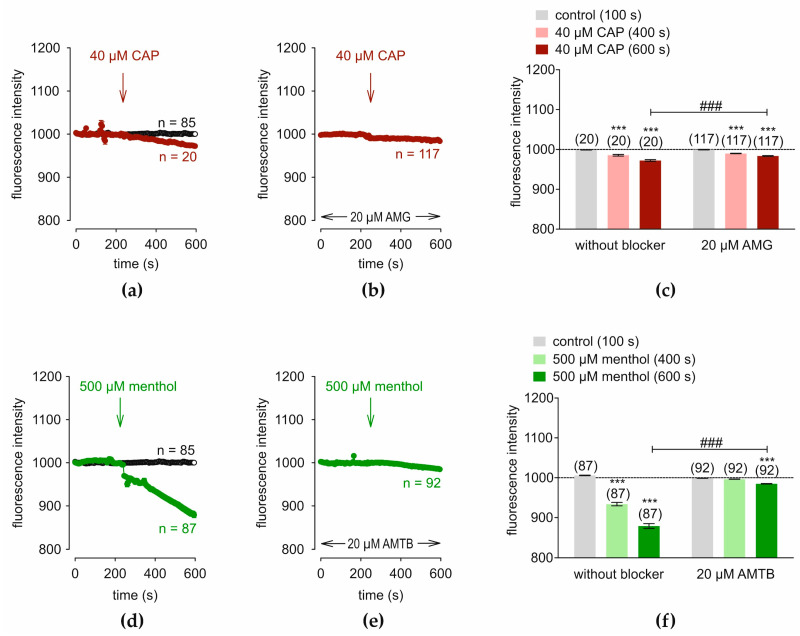
CAP and menthol induce decreases in fluorescence corresponding to a cell shrinkage that are suppressed by AMG (TRPV1) and AMTB (TRPM8) in calcein-loaded IOBA-NHC cells, respectively. The time (240 s) at which the agonists are added is shown by arrows. (**a**) CAP (40 µM) slightly decreases the apparent cell volume (red filled circles; (*n =* 20). For the control, no changes in the apparent cell volume occurred (*n =* 85) (open circles). (**b**) AMG (20 µM; *n =* 117) suppresses the rise in fluorescence intensity that 40 µM CAP induces. (**c**) Summary, wherein CAP and AMG are shown at 100 s (control) (gray column), 400 s (CAP) (bright red column), and 600 s (CAP) (dark red column). The dashed line represents the baseline f494 fluorescence intensity (1000). The asterisks (*) indicate statistically significant differences with and without CAP (*n =* 25 to *n =* 64; *** *p* < 0.001; paired tested). The hashtags (#) denote unpaired data with and without AMG (### *p* < 0.001). (**d**–**f**): Same experimental sequence as shown in (**a**–**c**). Instead of CAP, 500 µM menthol is used (green filled circles or columns; *n =* 87) and instead of 20 µM AMG, 20 µM of AMTB is added (*n =* 92).

TRPV1 involvement was assessed based on the individual effects of CAP (40 µM) and 20 µM AMG 9810 (AMG). CAP slightly decreased the f494 fluorescence intensity from 998.7143 ± 0.3412 at 100 s (control) to 985.418 ± 2.0939 at 400 s to 972.1538 ± 2.1628 at 600 s (*n* = 20; *** *p* < 0.001; paired tested) (Figure 5a). Subsequently, preincubation with 20 µM AMG and calcein (1 µM) simultaneously for 20 to 40 min suppressed the CAP-induced small shrinkage (Figure 5b). The f494 fluorescence intensity with AMG fell from 989.7317 ± 0.6851 at 400 s to 983.5000 ± 1.0233 at 600 s, respectively (*n* = 117; *** *p* < 0.001; paired tested) (Figure 5c). The AMG trace has a slightly lower negative slope than the trace with CAP (*n* = 20–117; ### *p* < 0.001; unpaired tested) indicating an inhibitory effect of AMG (Figure 5a,b). TRPM8 also mediates regulatory volume control since menthol (500 µM) decreased the f494 fluorescence intensity from 1005.7600 ± 0.6703 at 100 s (control) to 933.7168 ± 4.2768 at 400 s to 878.8047 ± 5.9864 at 600 s (*n* = 87; *** *p* < 0.001; paired tested) (Figure 5d). The effect of the antagonist AMTB (20 µM) on regulatory cell volume was apparent since it completely abolished the fluorescence intensity decline, which only declined from 998.6553 ±0.2153 at 100 s (control) to 996.2674 ± 0.3390 at 400 s and to 984.6566 ± 0.7036 at 600 s, respectively (*n* = 92; *** *p* < 0.001; paired tested) (Figure 5e,f). The AMTB trace has a considerably less negative slope than the trace with menthol demonstrating a strong inhibitory effect by AMTB (*n* = 87–92; ### *p* < 0.001; unpaired tested) (Figure 5f). Therefore, both TRPV1 and TRPM8 mediate regulatory cell volume responses to osmotic challenges.

### 3.6. NGF Induces Changes in Regulatory Cell Volume via TRPV1 and TRPM8

**Figure 6:**
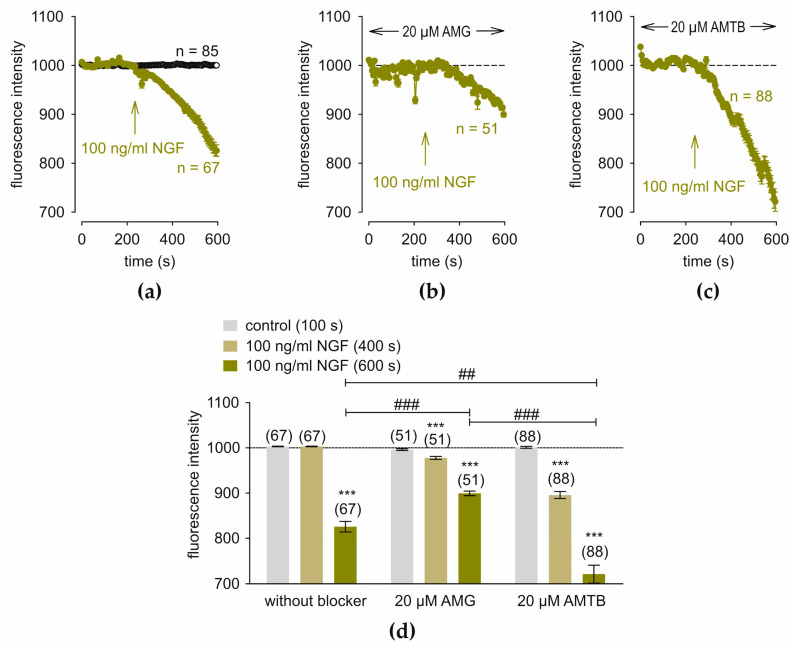
AMG suppresses NGF-induced cell shrinkage whereas AMTB increases this decline. The arrows indicate the time of the extracellular application of NGF at 240 s. (**a**) 100 ng/mL decreased the cell volume (*n* = 67; dark yellow filled circles). As control, there is no change in cell volume regulation in RLS without NGF application (*n* = 85) (open circles). (**b**) Partial TRPV1 blocking effect of 20 µM AMG on NGF-induced cell shrinkage (negative control) (*n* = 51). (**c**) AMTB augmented the NGF-induced cell shrinkage (*n* = 88). (**d**) Summary of the NGF-induced cell shrinkage with and without AMG or AMTB (both 20 µM). Data are shown at 100 s (control) (gray column), 400 s (NGF) (yellow column), and 600 s (NGF) (dark yellow column). The asterisks (*) indicate statistically significant differences with and without NGF (*n* = 51–88; *** *p* < 0.001; paired tested). The hashtags (#) refer to unpaired data with and without AMG or AMTB (*n* = 51–88; ### *p* < 0.001; ## *p* < 0.01; unpaired tested).

NGF (100 ng/mL) decreased the fluorescence intensity from 1003.0090 ± 0.6789 at 100 s (control) to 825.7206 ± 11.7042 at 600 s (*n* = 67; *** *p* < 0.001; paired tested) (Figure 6a). AMG partially suppressed this decline to 899.2784 ± 5.0839 at 600 s (*n* = 51–67; ### *p* < 0.001; unpaired tested), whereas AMTB augmented the apparent cell volume shrinkagesince the fluorescence intensity further decreased to 721.2696 ± 19.6394 at 600 s; *n* = 67–88; ## *p* < 0.01; unpaired tested) (Figure 6c,d). Taken together, blockage of TRPV1 partially suppresses the NGF-induced cell shrinkage, whereas TRPM8 blockage augments the NGF-induced decline. 

## 4. Discussion

IOBA-NHC is a stable cell line and an appropriate cell model for human conjunctival epithelial cells (HCjECs). It expresses TRPV1 and TRPM8, which corresponds to results obtained in: (1) in vitro electrophysiological assays; (2) RT-PCR; (3) immunohistochemistry of primary cultivated IOBA-NHC cells; (4) ex vivo human conjunctivas as well as pterygial cells [26,44,47,59,60,61]. Such pathology is associated with aberrant cell overgrowth of the IOBA-NHC cells [62]. These channels undergo stable functional expression since their channel expression is reproducible at different cell passages. Capsazepine (CPZ) has been used as a TRPV1 blocker [55]. However, it also blocks TRPM8 channels [63,64]. We used instead AMG 9810 since it is a more selective TRPV1 antagonist [56]. Regarding TRPM8, BCTC is instead often used but it also suppresses TRPV1 [63,65]. Therefore, we used AMTB as a selective TRPM8 blocker [57,58,66]. In IOBA-NHC cells and in human corneal epithelial cells (HCE-T), we showed that activation of TRPV1 channels leads to the release of pro-inflammatory cytokines [44,67]. TRPM8 also plays a role as it interacts with TRPV1 [54,68] and its activation can affect secretions by the lacrimal glands and meibomian gland cells [69]. Therefore, TRPV1 and TRPM8 modulation is being evaluated for its use as a therapy against dry eye disease (DED) [34,46,70,71,72].

Regarding NGF-mediated calcium regulation, Jiang et al. characterized how neurotrophins stimulate calcium uptake in mouse embryonic fibroblasts (NIH 3T3), which were transfected with the NGF receptor TrkA (high affinity) or the NGF receptor p75^NGFR^ (low affinity) [73]. NGF (100 ng/mL) induced an increase in calcium uptake provided the high-affinity NGF receptor was expressed. In this study, fura-2/AM was used [52] instead of fluo-3/AM because fura-2/AM detects smaller intracellular Ca^2+^ changes. With nifedipine (10 µM), a blocker of L-type Ca^2+^ channels, blocked NGF-induced Ca^2+^ influx [73]. We used a similar approach with NGF at the same concentration. NGF (100–250 ng/mL) induced a slight increase in intracellular Ca^2+^ (Figure 2a). With 250 ng/mL NGF, a slightly larger effect occurred (Figure S1), but it was still considerably smaller than the effects of either CAP or menthol. This smallness may be either attributable to activation of a low-affinity NGF receptor or a low expression of a high-affinity NGF TrkA receptor. Since TRPs like TRPV1 and TRPM8, as well as NGF receptors (TrkA), are (over)expressed in tumors such as uveal melanoma (UM) or retinoblastoma [74,75,76,77], we tested NGF on calcium regulation in human UM cells for comparison purposes. The NGF-induced Ca^2+^ influx is at a higher level than that in (healthy) IOBA-NHC cells (Figure S2). In contrast, AMG and AMTB fully suppressed the NGF-induced Ca^2+^ increase down to the baseline level (dashed line) in both cases (Figure 2b,c). Therefore, both TRPV1 and TRPM8 channel activation is involved in the NGF-induced Ca^2+^ increase. In contrast to the voltage-dependent L-type Ca^2+^ channels, TRPs are non-selective cation channels that are independently activated regardless of changes in membrane voltage, e.g., via changes in either temperature, pH, osmolarity, or pharmacologically [78,79,80,81]. Furthermore, epithelial cells have a resting membrane potential (RMP) of around −40 mV (e.g., in retinal epithelial cells) [82]. At this RMP, L-type channels cannot be significantly activated. Overall, the role of NGF in IOBA-NHC cells is complex and coupled to TRPV1 and TRPM8. Possibly certain ambient conditions may also induce NGF to trigger both TRPV1 and TRPM8 channel activation.

Passive store depletion of intracellular calcium by dialysis of the cells with 1 mM EGTA in a Ca^2+^-free solution followed the addition of external Ca^2+^. NGF triggered a transient SOC activation which induced a large Ca^2+^ transient. This response is similar to the effect of anaphylatoxin C5a in IOBA-NHC cells wherein an extremely large Ca^2+^ influx occurred followed by return to a baseline level (Figure 3a; dashed line) [51], which also occurred in tumor cells [51,83]. Since the Ca^2+^ store filling state controls the expression of TRPC1, TRPC3, and TRPV6 channels [84], it is possible that such TRPs are also expressed in IOBA-NHC cells. In particular, TRPV6 channels as Ca^2+^ sensors are highly Ca^2+^ selective and may explain the large Ca^2+^ influx. Notable, this response was amplified with NGF, but there are no studies describing NGF’s direct influence on TRPV6. However, a possible interaction between NGF and TRPV6 activity cannot be excluded and could be related to the role of NGF in cellular signaling, whereby TRPV6 downregulation impaired cellular Ca^2+^ influx and cell proliferation [85]. A possible explanation for this difference in Ca^2+^ responses might be that NGF also triggers other TRPs besides TRPV1 and TRPM8. Our protocol induced passive store depletion, as has been used in many studies to activate TRPV6 [85,86,87,88,89]. TRPC1 and TRPV6 may be molecular candidates for endogenous SOCs. Similarly, activation of TRPV6 channels is strongly dependent on the cytosolic-free calcium concentration in prostate tumor cells [90]. Lowering the intracellular-free Ca^2+^ concentration results in calcium influx, and the Ca^2+^ amplitude correlates with the degree of intracellular Ca^2+^ depletion achieved with different intracellular EGTA (or BAPTA) concentrations [91]. Overall, not only TRPV1 and TRPM8 appear to be modulated via NGF, but also SOC and other TRP channel subtypes may also be involved.

Regarding hyper- and hypotonic challenge, the responses to the anisosmotic challenges in the control experiments confirm both cell shrinkage and cell-swelling responses in IOBA-NHC cells. The effect of hypertonicity is relevant to dry eye diagnosis since (mild) tear film hyperosmolarity is a hallmark of this disease [6,7,92,93]. Shrinkage of the IOBA-NHC cells occurs in response to a hypertonic challenge, whereas osmoprotection is rendered through suppression of hypertonic-induced TRPV1 activation [47]. Inversely, an increase in the cell volume under a hypotonic challenge occurred in HCECs [94] and lung epithelial cells [95]. Troiano and Monaco evaluated the short-term effects of two kinds of artificial tears, both containing 0.4% hyaluronic acid with an osmolality of either 300 or 150 mosmol/L on the typical symptoms of patients suffering from dry eye disease and on the vitality of corneal and conjunctival epithelial cells [96]. They concluded that their hypotonic solution is effective in reducing dry eye symptoms since it decreased the tear film osmolality and improved the vitality of the epithelial cells in the cornea and conjunctiva [96]. Therefore, in the context of ocular surface health, hypotonic solutions can help to maintain the integrity and function of ocular surface epithelial cells. Another study investigating the efficacy of 0.18% hypotonic sodium hyaluronate (SH) eyedrops in a mouse model of experimental dry eye found that hypotonic SH eyedrops are more effective in improving ocular surface irregularity, reducing corneal staining and decreasing inflammatory cytokines, chemokine levels, and cells on the ocular surface than isotonic 0.5% carboxymethycellulose [97]. Corneal surface parameters can be improved by hypotonic 0.18% SH in their animal model. This can be partly explained by the intrinsic and dose-dependent acceleration of wound healing by SH. [97].

TRPV1 activation via CAP resulted in a marginal apparent cell volume shrinkage, which was blocked by AMG in both IOBA-NHC cells and HCE-T cells [47,54]. In contrast, activation of TRPM8 with menthol induced instead a larger shrinkage than CAP. This difference contradicts findings showing that TRPM8 is only as osmosensitive as TRPV1 (and TRPV4). Quallo et al. identified TRPM8 as a peripheral osmosensor responsible for the regulation of normal eye-blinking in mice [42]. Specifically, hyperosmotic responses were abolished by TRPM8 antagonists, such as AMTB (30 µM) and BCTC (3 µM), and were absent in dorsal root ganglia and trigeminal ganglion neurons isolated from TRPM8^(−/−)^ knockout mice [42]. Furthermore, Ca^2+^ imaging results of the effects of exposure to hyperosmolar sucrose caused Ca^2+^ transients, which were blocked by AMTB. These results provide evidence that TRPM8 channels act as a hyperosmosensor, and sweet foods can trigger pain (dentin hypersensitivity) [98,99]. In this context, Genova et al. showed with a different experimental approach in context with cell migration that a shrinkage was induced by the TRPM8 agonist icilin in endothelial cells, which are non-excitable cells like IOBA-NHC cells [100]. In conclusion, our results agree with the few other studies showing that TRPM8 activation induces cell shrinkage.

**Figure 7:**
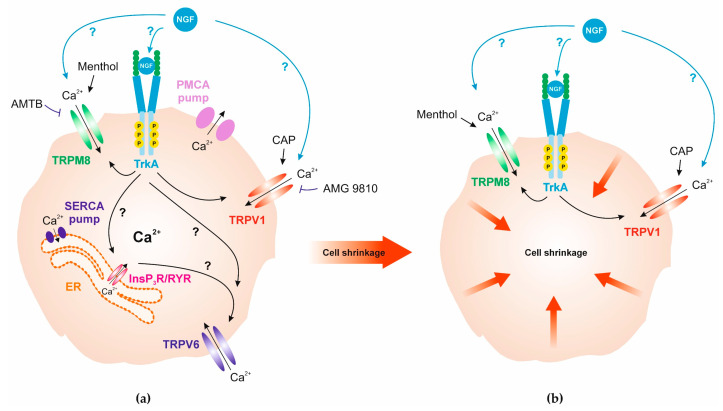
Simplified summary scheme illustrates functional TRP channel expression and Ca^2+^ homeostasis parameters that trigger cell shrinkage in IOBA-NHC cells. TrkA = Tropomyosin receptor kinase A, SERCA = Sarcoplasmic/endoplasmic reticulum Ca^2+^-ATPase, PMCA = Plasma membrane Ca^2+^-ATPase; ER = endoplasmic reticulum, InsP_3_R = Inositol 1,4,5-trisphosphate receptor, RYR = Ryanodine receptor. (**a**) Whereas TRPV1 is activated by CAP [55] and blocked by AMG 9810 [56] (Figure 1a,b), TRPM8 is activated by menthol [40] and blocked by AMTB [58] (Figure 1d,e). The epithelial Ca^2+^ channel TRPV6 is a Ca^2+^ sensor that is a type of SOC and activated either by active or passive Ca^2+^ store depletion. Active depletion involves either InsP_3_ or ryanodine receptors. TRPV6 can also be activated by passive store depletion without involving InsP_3_ or ryanodine receptors (as in this study) (Figure S3) [91,106,107,108]. (**b**) NGF application binds to TrkA and leads to a Ca^2+^ increase via TRPV1 activation (Figure 2b) and TRPM8 activation (Figure 2c). Activation of TRPV1 by CAP and TRPM8 by menthol, as well as extracellular application of NGF leads to cell shrinkage (Figure 5a,d and Figure 6a).

TRPV1 and TRPM8 involvement in mediating cell volume responses to NGF show that NGF induces large decreases in cell volume (Figure 7). In pheochromocytoma cells (PC12), changes in cell size and volume correlate with the cell growth program induced by NGF [101]. In addition, Leung et al. showed that NGF increases the apparent cell volume by increasing cotransport activity and sensitivity in PC12 cells [102]. On the other hand, Tandrup et al. studied shrinkage of dorsal root ganglion cells by NGF, and they showed that this response is related to an apoptotic process [103]. We found that the responses to either AMG or AMTB were complex. While TRPV1 blockage partially suppressed shrinkage to NGF, it was particularly pronounced subsequent to TRPM8 blockage. This larger shrinkage induced by TRPM8 blockage is not proportional to the larger difference in the magnitude of the Ca^2+^ transients induced by CAP than NGF and the larger changes in Ca^2+^ levels, in which both AMG and AMTB completely blocked the NGF-induced calcium influx. A possible explanation for this difference might be that both NGF and AMTB are also responsive to cell stress and cell apoptosis in a non-physiological environment since induction of cell shrinkage is a morphological hallmark of apoptosis. Bortner et al. named it apoptotic volume decrease (AVD) and reviewed the role of cell shrinkage and monovalent cations in apoptosis [104]. These types of stresses may also induce changes in potassium and sodium fluxes that also activate TRPs. This is a possibility since they are non-selective cation channels whose activation may increase the influx of other cations besides Ca^2+^ [105].

Potassium and chloride channels have also been described in connection with apoptotic cell shrinkage and apoptosis in cortical neurons [109,110]. Taken together, TRPV1 activation via CAP and TRPM8 activation via menthol, as well as application of NGF, induced cell shrinkage (Figure 5a,d, Figure 6a, and Figure 7). TRPV1 blockage partially suppressed the NGF-induced cell shrinkage (Figure 6b), whereas the TRPM8 blockage had a larger effect leading to a disproportionately larger decrease in apparent cell volume (Figure 6c,d). Therefore, NGF induces cell volume regulatory responses through the activation of TRPV1 and TRPM8 channels, whereas blockage of TRPM8 activation by a hypertonic challenge induces a larger shrinkage response to such stress even though the TRPV1-induced Ca^2+^ transient rise and its subsequent decline are larger than those induced by TRPM8 activation. It may be that this disconnection is ascribable to differences in permeation of other divalent cations besides Ca^2+^ through the non-selective TRP ion channels.

### 4.1. Clinical Implications

Recombinant NGF has been licensed and is already used in clinical treatment of neurotrophic keratopathy [111]; other promising clinical applications include treatment of glaucoma [112] and retinitis pigmentosa [113]. Our findings will likely contribute to a more detailed understanding of how NGF’s effects on ocular surface health and disease may be improved. NGF induces regulatory volume responses to hypertonic and hypotonic stress through joint modulation of TRPV1 and TRPM8 ionic channel activity that in turn induce transient Ca^2+^ signaling activity that triggers changes in cell volume. We found that in response to a hypotonic challenge, the sole activation of TRPV1 facilitates restoration of isotonic cell volume during exposure to this stress. On the other hand, TRPM8 activation has effects that counter the restorative effects of TRPV1 activation on cell volume. Accordingly, it is conceivable that in a clinical setting the restorative effect on maladaptive cell swelling may be improved through jointly applying a selective TRPM8 antagonist with NGF. This type of insight is also pertinent for countering the pathological effects of a hypertonic tear film that can be encountered in patients afflicted with neurotrophic keratopathy. In these individuals, a hypertonic tear film induces cell volume shrinkage, which can counter maintenance of corneal epithelial barrier function. Disruption of this function can lead to pathogen stromal infiltration and chronic inflammation if restoration of isotonic cell volume is delayed. To overcome this hindrance, it may be possible to selectively activate TRP channel behavior favoring increases in net ionic transport activity that optimize restoration of isotonic cell volume despite the offsetting effect of a hypertonic challenge resulting from an increase in tear film osmolarity in these patients. It is notable that plasma levels of NGF are significantly higher in patients with vernal keratoconjunctivitis compared to healthy controls [114] whereby NGF modulates the synthesis of substance P (SP), a neuropeptide involved in the pathogenesis of human allergic diseases [115]. Additionally, tear NGF levels are also elevated in patients suffering from keratoconjunctivitis sicca, which can be reduced by 0.1% prednisolone treatment [116]. This study shows that NGF cell volume behavior responses are mediated through the joint activation of TRPV1 and TRPM8 (Figure 2, Figure 6, and Figure 7). Such an association also exists at the neuronal level [117,118,119]. The goal of dry eye treatment is to somehow suppress both the elevated NGF levels and the dry eye symptomology in order to reduce the rises in inflammatory parameters (pro-inflammatory cytokine release), resulting from TRPV1 activation [67]. Interestingly, blocking TRPV1 activity also suppressed both the NGF-induced calcium increase and the NGF-induced cell shrinkage. Blocking TRPM8 enhanced the NGF-induced cell shrinkage more than blockage of TRPV1 activation. The larger hypertonic-induced increase in cell shrinkage induced by blockage of TRPM8 may account for its usage in promoting a positive clinical effect. For example, Borneol activates TRPM8 channels, and it modestly increases ocular surface wetness. This makes it particularly useful in treating dry eye syndrome [120].

### 4.2. Limitations of This Study

It should be noted that our study has certain technical limitations. Fura-2 fluorescence imaging was used to measure very small changes in intracellular calcium concentration [52]. However, fura-2 fluorescence imaging has several limitations that can lead to incomplete or imprecise data interpretation. Calcium signaling often occurs in localized regions within the cell, such as near ion channels or in intracellular organelles like the endoplasmic reticulum. Fura-2, being a bulk indicator, cannot resolve these fine spatial gradients. Thus, it may not detect any differences in the calcium dynamics at specific cellular sites (e.g., near the plasma membrane or in subcellular compartments). Furthermore, the kinetics of calcium influx and efflux (especially in very rapid signaling events like in neuronal or muscle cells) might be averaged out, which prevents detection of transient, high-frequency calcium spikes. However, this limitation is not pertinent to our study because the tissue is non-excitable. Another limitation is that we performed qualitative measurements instead of quantitative measurements of intracellular calcium concentration in the nM range. Therefore, the Ca^2+^ changes were reported as relative changes in the f340/f380 fluorescence ratios. However, if the calibrations were used to obtain quantitative analyses, the results would still coincide with the calcium response patterns because only the scaling of the Y-axis would change. Another limitation is that the fluorescence signal can become nonlinear at very high or very low calcium concentrations, which can lead to errors in quantifying calcium levels, particularly if the dye is over-saturated or under-saturated. Nevertheless, fura-2 fluorescence calcium imaging provides an effective and widely used method for observing calcium signaling, especially for detecting relatively large, systemic changes in calcium levels. The rationale for using fura-2 is that it allows for real-time monitoring of calcium dynamics in living cells without needing to extract or destroy the cells. Furthermore, fura-2 has a high sensitivity to changes in calcium concentration, making it useful for detecting even small variations in calcium levels, which is crucial for studying cellular signaling processes. Finally, it can be applied to a wide range of cell types, including neuronal, muscle, endothelial, and epithelial cells.

In addition, fluorescence calcein imaging was used to measure fluorescence intensity responses, which approximately correlate proportionally with changes in the cell surface and apparent cell volume that are induced by either a hypertonic or hypoosmotic stress [121,122]. Technical limitations of light-sensitive fluorescent dyes include bleaching effects, which become more pronounced during extended periods of dye loading of the cells. This limitation is evident if imaging measurements obtained under identical conditions are compared from different cell populations loaded for varying times. In this case, the fluorescence responses may not be reproducible. Accordingly, the cells with the most intense staining also elicit larger fluorescence signals and thus they undergo more bleaching than the less intensely dye stained cells (negative slope in the graph). In this study, however, this confounding effect was corrected in two ways. Firstly, the evaluation software TIDA for Windows (HEKA Electronic, Lamprecht, Germany) was used to perform a so-called drift correction. It had to be considered that the fluorescence signal under control conditions needs about 1–2 min for an adaptive adjustment until the baseline stabilizes. This is an extremely sensitive parameter which, if ignored, can lead to distortion of the measurement curves in the worst case. Secondly, it is recommended to try not to load the cells for too long a period with the fluorescent dye. However, this has the disadvantage that the signal-to-noise ratio becomes less favorable (more data scatter). In addition, manually adding (pipetting) the drugs into the test solutions can lead to pipetting artifacts. These errors can also be eliminated by the aforementioned TIDA software. There are also biological limitations. One such limitation is cell stress and non-physiological conditions during the measurements (room temperature, no CO_2_, etc.), especially during the transfer of the cells on a coverslip from a Petri dish into the bath chamber. Cells suffer considerably from the evaporative effect that occurs within a few seconds during transfer. Only a 30 s delay in performing the transfer is long enough for the cells to undergo apoptosis due to Ca^2+^ overload (calcium apoptosis link) [123]. Another biological limitation is the use of the spontaneously immortalized IOBA-NHC cell line, which can change its genotype and phenotype over time due to mutation and selection [124]. Therefore, care must be taken when interpreting the results as cell lines do not always accurately replicate the primary cell phenotype [124]. In our study, we used the IOBA-NHC cell line as a well-accepted model of the native human conjunctival epithelium [50]. Furthermore, the results and the phenotype of the IOBA-NHC cell line were confirmed to correspond with primary cultivated IOBA-NHC cells [60]. Furthermore, we tested the electrophysiological characteristics of the IOBA-NHC cell line several times for functional expression of TRPV1 and TRPM8 at different cell passages for more than 10 years. It is evident that at least the calcium response patterns have not changed significantly during this time period [44,47].

## 5. Conclusions

TRPV1 and TRPM8 are functionally expressed in IOBA-NHC cells, which is a relevant model of HCjE. This correspondence confirms the results of previous studies and their TRPV1 and TRPM8 functional stability. Passive store depletion is a protocol for assessing the presence of SOC activity involving other TRPs, such as TRPV6. With NGF, a larger transient store-operated Ca^2+^ channel activation induced an increase in the Ca^2+^ level lasting several minutes, suggesting that hypertonic stress amplifies the response to NGF, which in turn promotes larger activation of the TRPV1 and TRPM8 channels. NGF alone under isotonic conditions also increased intracellular Ca^2+^ in IOBA-NHC cells, but at a relatively low level compared to the larger Ca^2+^ influxes resulting from the activation of other TRP channel subtypes. Since the NGF-induced Ca^2+^ increase could be suppressed by the blockage of either TRPV1 or TRPM8, crosstalk between NGF and these TRPs may account for this effect. Moreover, NGF by itself induced a much larger shrinkage response than that induced by TRPV1 activation. However, the NGF-induced cell shrinkage could be suppressed by blockage of TRPV1, whereas blockage of TRPM8 failed to suppress the NGF-induced cell shrinkage, indicating a complex type of regulation between intracellular Ca^2+^ regulation and cell volume regulation in IOBA-NHC cells.

## Data Availability

The data presented in this study are available on request from the corresponding author.

## Supplementary Materials

The following supporting information can be downloaded at: https://www.mdpi.com/article/10.3390/cells14100719/s1, Figure S1: NGF increased intracellular Ca^2+^. The time dependent changes are shown as relative intracellular Ca^2+^ levels in fura2-loaded IOBA-NHC cells; Figure S2: Capsazepine (CPZ) suppresses NGF-induced Ca^2+^ rises; Figure S3: Ca^2+^ entry after passive store depletion in IOBA-NHC cells (IOBA-NHC cells).
