# Supplementary material for: Nerve Growth Factor Modulates Regulatory Cell Volume Behavior via Stimulating TRPV1, TRPM8 Channels and Inducing Ca2+ Signaling in Human Conjunctival Epithelial Cells
Source: Cells. 2025 May 15;14(10):719. doi: 10.3390/cells14100719 (PMC12109909; doi:10.3390/cells14100719)
Supplement: Supplementary file 1 [file cells-14-00719-s001.zip › cells-3598471-supplementary.pdf]

cells

MDPI

Supplementary Materials:

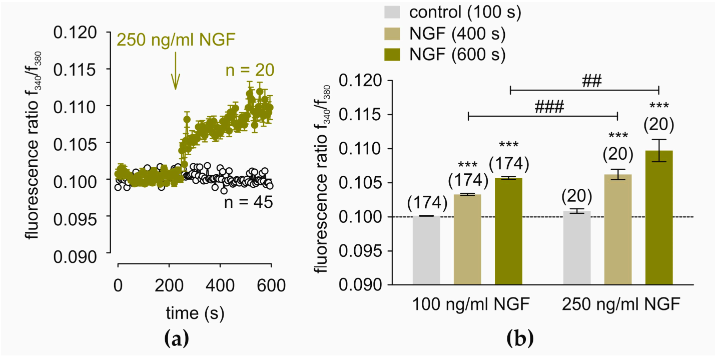

Figure S1: NGF increased intracellular Ca^{2+}. The time dependent changes are shown as relative intracellular Ca^{2+} levels in fura2-loaded IOBA-NHC cells. The number (*n*) indicates the number of cells examined in this set of experiments. The arrows denote the point of time of the extracellular application of NGF (250 ng/ml) at 240 s. **(a)** 250 ng/ml induces increases in Ca^{2+} entry (*n* = 20; dark yellow filled circles). As control without NGF application, no changes in intracellular Ca^{2+} level could be observed (*n* = 45) (open circles). **(b)** Statistical analyses of the NGF- induced Ca^{2+} response patterns at two different concentrations (100 vs 250 ng/ml). Columns represent mean values ± SEM of the fluorescence ratio at 100 s (control) (gray column), 400 s (NGF) (yellow column) and 600 s (NGF) (dark yellow column). The dashed line represents the reference line for baseline value (0.1). The asterisks (*) indicate statistically significant differences without and with 250 ng/ml NGF (*n* = 20 - 174; ****p* < 0.001; paired tested). The hashtags (#) refer to unpaired data with the two different NGF concentrations (*n* = 20 - 174; ###*p* < 0.001; ##*p* < 0.01; unpaired tested).

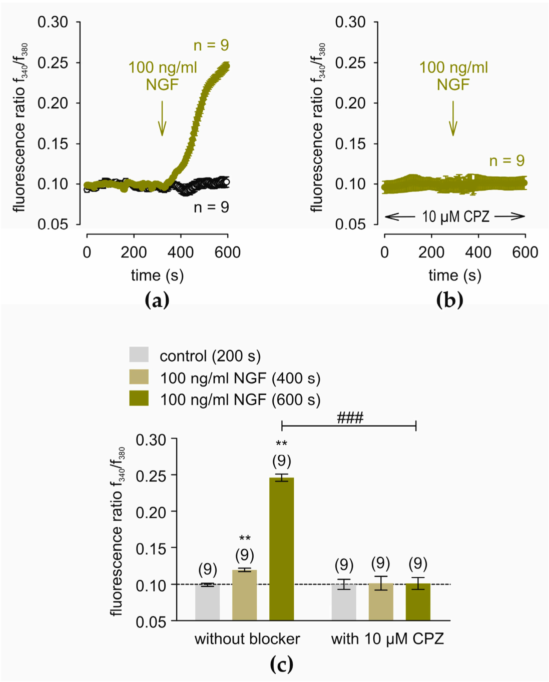

Figure S2: Capsazepine (CPZ) suppresses NGF-induced Ca^{2+} rises. The time dependent changes are shown as relative intracellular Ca^{2+} levels in fura2-loaded UM 92.1 cells. The number (*n*) indicates the number of cells examined in this set of experiments. The dashed line represents the reference line for baseline value (0.1). The arrows indicate the point of time of the extracellular application of NGF at 32 s. **(a)** 100 ng/ml induces clear increases in Ca^{2+} entry (*n* = 9; dark yellow filled circles). As

control without NGF application, no changes in intracellular Ca^{2+} level could be observed (n = 9) (open circles). **(b)** Representative graph of the TRPV1 blocking effect of 10 μM CPZ on NGF-induced Ca^{2+} influx (negative control) (n = 9). **(c)** Statistical analyses of the NGF- induced Ca^{2+} response patterns with and without CPZ (10 μM). Columns represent mean values ± SEM of the fluorescence ratio at 100 s (control) (gray column), 400 s (NGF) (yellow column) and 600 s (NGF) (dark yellow column). The asterisks (*) indicate statistically significant differences with and without NGF (n = 9; ** $p < 0.01$; paired tested). The hashtags (#) refer to unpaired data with and without CPZ (n = 9; ### p < 0.001; unpaired tested).

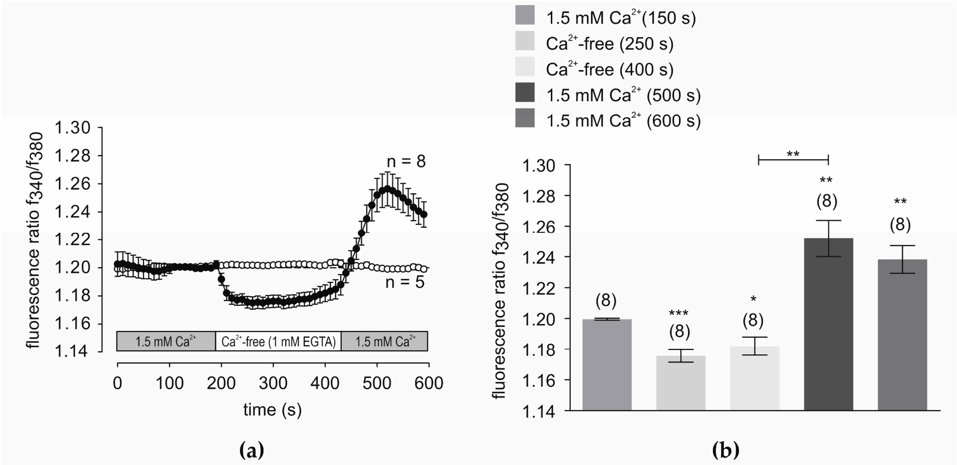

Figure S3: Ca^{2+} entry after passive store depletion in IOBA-NHC cells (IOBA-NHC cells). It should be noted that these measurements were carried out with the photomultiplier fluorescence calcium recording system mentioned in the appendix. **Data are means ± SEM.** The number (n) indicates the number of coverslips used in this set of experiments, where each coverslip contained 20 – 30 cells. **A control measurement for 10 min was carried out (control base line) (open circles) n = 5 coverslips. From 180 s, the solution was changed from a 1.5 mM** Ca^{2+} **containing solution (gray bar below the trace) to a** Ca^{2+} **free solution with 1 mM EGTA (white bar below the trace).** From 420 s, the Ca^{2+} **free solution was again changed back to the control solution containing 1.5 mM** Ca^{2+} (gray bar below the trace)**. (a)** In absence of extracellular Ca^{2+}, the Ca^{2+} decreased below the baseline level. There was a Ca2+ transient above the baseline when external 1.5 mM Ca^{2+} was added (RLS) (n = 8 coverslips, filled circles). **(b)** Summary of the experiment with IOBA-NHC cells with and without extracellular Ca^{2+}. The asterisks (**) designate significant decrease in Ca^{2+}-free RLS (t = 250 s, 400 s; n = 8 coverslips; ** $p < 0.01$ at the minimum; paired tested) compared to control (t = 150 s) as well as an increase in Ca^{2+} in the presence of extracellular Ca^{2+} (t = 500 s, 600 s; n = 8 coverslips; ** $p < 0.01$; paired tested) compared to control (t = 150 s).

Appendix

Materials and Method concerning supplementary materials

Materials

CPZ and icilin were procured from Cayman Chemical Company (Ann Arbor, Michigan, U.S.A.). Medium and supplements for cell culture were ordered from Life Technologies Invitrogen (Karlsruhe, Germany) or Biochrom AG (Berlin, Germany). Melanocyte Growth Medium M2 was obtained from Promocell (Heidelberg, Germany). Dispase II was ordered from Boehringer (Ingelheim, Germany) and accutase was provided by PAA Laboratories (Pasching, Austria). Unless otherwise stated, all other reagents were procured from Sigma (Deisenhofen, Germany).

Cell culture

Regarding Fig. S3, the uveal melanoma cell line 92.1 (UM 92.1) was used, which was kindly provided by Martine Jager and colleagues (Leiden University; Netherlands) (De Waard-Siebinga, I.; Blom, D.J.; Griffioen, M.; Schrier, P.I.; Hoogendoorn, E.; Beverstock, G.; Danen, E.H.; Jager, M.J. Establishment and characterization of an uveal-melanoma cell line. *Int J Cancer* **1995**, *62*, 155-161). In brief, UM cells were grown in RPMI-1640 supplemented with 10% fetal bovine serum (FBS), 4 mM L-glutamine, penicillin/streptomycin at 37°C under 10% CO_2 atmosphere and 80% humidity (Mergler, S.; Derckx, R.; Reinach, P.S.; Garreis, F.; Bohm, A.; Schmelzer, L.; Skosyrski, S.; Ramesh, N.; Abdelmessih, S.; Polat, O.K.; et al. Calcium regulation by temperature-sensitive transient receptor potential channels in human uveal melanoma cells. *Cell Signal* **2014**, *26*, 56-69, doi:10.1016/j.cellsig.2013.09.017).

Photomultiplier fluorescence calcium recording

Time-dependent changes of free intracellular calcium concentration ($[Ca^{2+}]_i$) were recorded with the fluorescent dye fura-2/AM. IOBA-NHC cells were pre-incubated with culture medium containing 2 µmol/l fura-2/AM for 15 – 45 minutes at 37 °C. Prior to the experiments, the cells were rinsed with RLS containing 150 mmol/l NaCl, 6 mmol/l CsCl, 1 mmol/l $MgCl_2$, 10 mmol/l glucose, 10 mmol/L HEPES and 1.5 mmol/l $CaCl_2$ at pH ~ 7.4 (~ 300 mosmol/L) to remove any dead cells or cellular debris. Fura-2 fluorescence in the washed cells was measured at room temperature using a digital imaging system (TILL Photonics, Munich, Germany) in connection with a detector (Hamamatsu Photonics, Sunayamacho, Nakaku, Hamamatsu City, Shizuoka Pref., Japan) and a fluorescence microscope (BW50I, Olympus, Hamburg, Germany). Fura-2 fluorescence response signals were alternately excited at 340 and 380 nm wavelength and emission were detected from cell clusters every 500 ms at 510 nm. For the diagram, data points are shown in 10 s steps. The fluorescence ratio (f340nm/f380nm) was calculated as a relative index of changes in $[Ca^{2+}]_i$. Results are shown as mean traces of the fluorescence ratio f340nm/f380nm ± SEM with *n*-values indicating the number of experiments (coverslips) per data point.
